# Effect of ultrasonic activation on setting time, pH and calcium ion release, solubility, and chemical structure of calcium silicate sealers

**DOI:** 10.1590/0103-6440202405824

**Published:** 2024-07-22

**Authors:** Simone Argenta Scalabrin, Lina Naomi Hashizume, Theodoro Weissheimer, Gabriel Barcelos Só, Jefferson Ricardo Pereira, Milton Carlos Kuga, Ricardo Abreu da Rosa, Marcus Vinicius Reis Só

**Affiliations:** 1 Department of Conservative Dentistry, School of Dentistry, Rio Grande do Sul Federal University (UFRGS), Porto Alegre, RS, Brazil; 2 Department of Prosthodontics, Unisul - Universidade do Sul de Santa Catarina, Tubarão, SC, Brazil; 3 Department of Restorative Dentistry, Araraquara Dental School, São Paulo State University,, Araraquara SP, Brazil.

**Keywords:** Calcium silicate, endodontics, physicochemical properties, spectroscopy, ultrasound

## Abstract

This study evaluated the setting time, pH, calcium ion release, solubility, and chemical structure of four calcium silicate sealers after ultrasonic activation (UA). Five sealers were evaluated: Sealer Plus (SP - control); Sealer Plus BC (SPBC), Bio C Sealers (BCS), Endosequence BC Sealer (EBC), and BioRoot RCS (BR). Ten groups were created based on the use or not of ultrasonic activation: SP; SP/UA; SPBC; SPBC/UA; BCS; BCS/UA; EBC; EBC/UA; BR; and BR/UA. Setting time was performed based on ISO 6876:2012 and ASTM C266-07 specifications. Solubility at 24hs, based on ISO 6876:2012. pH and calcium release were evaluated at 1, 24, 72, and 168hs. Raman spectroscopy was used to evaluate structural changes. Quantitative data were analyzed using One-Way ANOVA and Tukey post-hoc test (α=5%). Raman spectroscopy results were qualitatively analyzed. Setting times and solubility of all sealers were not affected by UA (p>0.05). The highest solubility was found for BCS, BCS/UA; and BR, BR/UA (p<0.05). After 24hs, calcium silicate sealers had higher pH than SP and SP/UA (p<0.05). BR and BR/UA had the highest pH at all time points. SP and SP/UA had stable pH at all time points. SP and SP/UA had the lowest calcium release values at all time points (p<0.05). EBC and EBC/UA calcium release significantly differ at 24,72 and 168hs (p<0.05). No chemical changes were observed during Raman spectroscopy. In conclusion, ultrasonic activation affected calcium ion release only for EndoSequence BC Sealer. Ultrasonic activation did not influence the initial and final setting time, solubility, pH, and chemical structure of any investigated sealers.

## Resumo

Esse estudo avaliou o tempo de presa, pH, liberação de íons cálcio, solubilidade e estrutura química de quatro cimentos à base de silicate de cálcio após a ativação ultrassônica (AU). Cinco cimentos foram avaliados: Sealer Plus (SP - control); Sealer Plus BC (SPBC), Bio C Sealers (BCS), Endosequence BC Sealer (EBC) e BioRoot RCS (BR). Dez grupos foram criados com base no uso ou não de ativação ultrassônica: SP; SP/UA; SPBC; SPBC/UA; BCS; BCS/UA; EBC; EBC/UA; BR; e BR/UA. Tempo de presa foi realizado baseado nas especificações ISO 6876:2012 e ASTM C266-07. Solubilidade em 24hs, baseado na ISO 6876:2012. pH e liberação de cálcio foram avaliados em 1, 24, 72 e 168hs. Espectroscopia Raman foi usada para avaliar alterações estruturais. Os dados quantitativos foram analisados utilizando ANOVA de uma via e teste post-hoc de Tukey (α=5%). Os resultados da espectroscopia Raman foram analisados qualitativamente. Os tempos de presa e a solubilidade de todos os cimentos não foram afetados pelo AU (p>0.05). Maior solubilidade foi encontrada para BCS, BCS/AU; e BR, BR/AU (p<0.05). Após 24hs, os cimentos de silicato de cálcio apresentaram pH mais elevado que SP e SP/AU (p<0.05). BR e BR/AU tiveram o pH mais alto em todos os momentos. SP e SP/AU apresentaram pH estável em todos os momentos. SP e SP/AU tiveram os menores valores de liberação de cálcio em todos os momentos (p<0.05). A liberação de cálcio EBC e EBC/AU diferiram significativamente em 24,72 e 168hs (p<0.05). Nenhuma alteração química foi observada durante a espectroscopia Raman. Em conclusão, a ativação ultrassônica afetou a liberação de íons cálcio apenas para o EndoSequence BC Sealer. A ativação ultrassônica não influenciou o tempo de presa inicial e final, a solubilidade, o pH e a estrutura química de nenhum dos cimentos investigados.

## Introduction

Endodontic sealers are essential for filling the areas between the dentin wall and gutta-percha after chemo-mechanical preparation, preventing the persistence of bacteria and recontamination of the canal ^1^. They should, therefore, have adequate physicochemical properties, such as dimensional stability, low solubility, dentinal wall adhesion, and appropriate working and setting time ^2^ ^,^ ^3^ ^,^ ^4^.

Calcium silicate-based sealers are mainly composed of calcium silicate, calcium phosphate, aluminum oxide, zirconia, bioactive glass, vitroceramic or hydroxyapatite ^5^ ^,^ ^6^. They are classified as bioinert, bioactive, or biodegradable, depending on their composition ^6^. Essentially, those containing calcium silicate are classified as bioactive, as they interact with surrounding tissues and produce a mineralized matrix ^6^.

More recently, it has been shown that ultrasonic activation (UA) of resin-based sealers promotes greater intratubular penetration, bond strength, and space-filling ^7^ ^,^ ^8^ ^,^ ^9^ ^,^ ^10^ ^,^ ^11^. Similar findings were obtained when evaluating bond strength and space formation between the obturation and the dentin wall after UA of calcium silicate sealers ^10^. However, calcium silicate sealers, when submitted to temperatures above 100^(^ C, undergo reversible changes in their chemical structure, and irreversibly lose the water in their composition. Therefore, microstructural changes may occur and affect their physicochemical properties. 

Since water plays an important role in calcium silicate sealers, and only a few evidence are available on physicochemical properties of calcium silicate-based sealers ^12^, this study aimed to evaluate the effects of ultrasonic activation on setting time, pH and calcium ion release, solubility and chemical structure of four calcium silicate-based sealers (Bio-C Sealer® - Angelus, Londrina, Brazil; Sealer Plus BC® - MK Life, Porto Alegre, Brazil; EndoSequence BC Sealer® - Brasseler USA, Savannah, GA; BioRoot RCS® - Septodont, Saint-Maur-des-Fosses, Cedex, France). The null hypothesis was that there would be no differences in setting time, pH and calcium ion release, solubility, and chemical structure with and/or without ultrasonic activation.

## Materials and methods

This study was an in vitro laboratory-based experiment approved by the Research Committee (COMPESQ) of the School of Dentistry of the Federal University of Rio Grande do Sul (UFRGS), Brazil.

### Investigated Sealers and Experimental Groups

Five sealers were used in the present study. Four calcium silicate-based sealers and one epoxy resin-based sealer (as control). Their chemical compositions are presented in Box 1.

Ten groups were formed according to the sealer and use of UA: 1) Sealer Plus without ultrasonic activation (SP); 2) Sealer Plus with ultrasonic activation (SP/UA); 3) Sealer Plus BC (SPBC); 4) SPBC/UA; 5) Bio C Sealer (BCS); 6) BCS/UA; 7) EndoSequence BC Sealer (EBC); 8) EBC/UA; 9) BioRoot RCS (BR); 10) BR/UA.

**Box 1:**
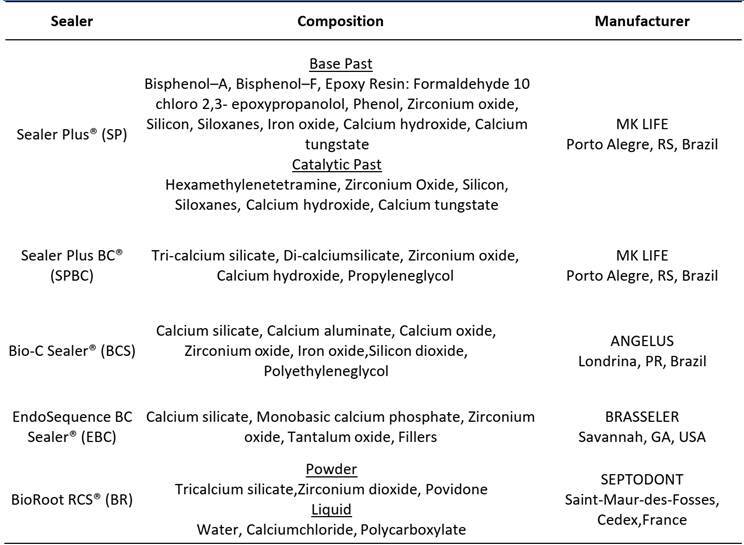
Composition of tested sealers

### Sample Size Calculation

Sample size calculation was performed using G*Power v3.1 software for Mac (Heinrich Heine Universität, Düsseldorf, Germany) by selecting the Student T test. Data from a previous study was used ^13^. The effect size for this study was established at 11.88. An alpha-type error of 0.05 and a beta power of 0.95 were used. A total of at least 3 samples per group was determined to detect significant differences.

### Samples Preparation

Polyethylene tubes, 10 mm in length, 1.0 mm in diameter, and with closed ends were weighed on an analytical scale (BEL Engenharia, Milan, Italy) to ensure consistency among samples. The sealers were then inserted into the tubes. Specimens in the UA experimental groups underwent activation for 60 seconds using a smooth ultrasonic insert with a tip diameter of 0.20mm and 0.10 mm taper (Irrisonic, Helse Dental Technology, São Paulo, Brazil) coupled to an ultrasound unit (Newtron Booster, Satelec Acteon, Merignac, France) calibrated at the power recommended by the manufacturer (15%).

### Evaluation of Initial and Final Setting Time

The initial and final setting times of the sealers were evaluated according to ISO 6876:2012 ^14^ and ASTM C266-07 specifications ^15^. Dental stone Type IV plaster casts (Durone IV Salmon; Dentsply, Petrópolis, Brazil), measuring 10 mm in diameter and 2 mm in height, were fabricated and immersed in distilled water for 24 hours at 37° C. 

Three samples per group (n=30), of 1mL each were produced. Samples from UA groups underwent activation. The molds were filled with the samples and kept in an oven at 37° C and 95% humidity. Thirty minutes later, a 100 g Gillmore needle (i:100 g/2-mm tip, ii:456 g/1-mm tip) was placed vertically on the surface of the samples. The procedure was repeated every 60 seconds until the sample surface was no longer marked, and this was defined as the initial setting time. 

The evaluation of the final setting time began shortly after the initial time was set. For this measurement, a 456.5 g Gillmore needle with a 1-mm active tip was placed vertically on the surface of the samples, and the same interval (60s) was used to determine the final setting time.

### Evaluation of Solubility

Three samples per group were prepared with 1 mL of each tested sealer and placed in polyethylene tubes. Fifteen samples underwent UA, and fifteen did not. The samples from each group were placed in plaster casts fabricated using dental stone Type IV (n=30; Durone IV Salmon; Dentsply, Petrópolis, Brazil). Each cast measured 10 mm in diameter and 2 mm in height, according to the ISO 6876:2012 specifications ^15^. 

A nylon wire was placed into the fresh sealer samples. The samples were then placed between two glass plates and wrapped in cellophane, and two wet pieces of gauze were placed between the molds and the glass, as previously described ^13^. These sets were kept in an oven at 37° C and 95% air humidity for a period three times longer than their setting time.

After that, the samples were removed from the molds, weighed three times on an analytical scale at an accuracy of 0.001g (Shimadzu, Tokyo, Japan), and placed in plastic tubes (Falcon, Labor Import, Osasco, Brazil) containing 50 mL of distilled water. The samples were kept for 24 hours in an oven at 37° C. The nylon wire was used to immerse the samples in distilled water without touching the walls of the bottle during the test.

After 24 hours, the samples were removed from the vials, dried in a dehumidifier for 24 hours, and weighed three times to obtain their final weight. Solubility values, calculated by determining the weight loss after immersion, were expressed as percentages.

### Evaluation of pH and Calcium Ion Release

Five samples per group/timepoint (n=200) were used for the pH and calcium ion release tests. The sealers were inserted into polyethylene tubes using 1 mL syringes. Twenty-five samples per timepoint were used in the groups that underwent UA; and twenty-five samples per timepoint, in the groups that did not. All samples were stored in vials containing 10 mL of deionized water and kept in an oven at 37^o^ C for posterior measurements. Five samples from each group were used for pH readings at 1, 24, 72, and 168 hours. 

Before pH readings, the sealers were removed from the vials, and the solutions were stirred for five seconds. pH was measured using a digital pH meter (Digimed DM-22, São Paulo, Brazil), calibrated using solutions at pH 4 and 7. 

Calcium ion release was determined by using the deionized water of the vials in which the samples were inserted. The same time points were evaluated using colorimetric spectroscopy with arsenazo III reagent (Merck KgaA, Darmstadt, Germany).

### Evaluation of Chemical Structure of Sealers

The chemical structure of the sealers with and without UA was analyzed using Raman spectroscopy (Senterra; Bruker Optics, Ettlingen, Germany). A small sample of each tested sealer (n=1) was placed on a glass plate, and one sample of each sealer underwent UA for 1 minute on the surface of the glass slab.

A laser diode beam with a wavelength of 785 nm, power of 100 mW, spectral resolution of approximately 3.5 cm-1, and fluorescence reduction filter was used for sample excitation, and the spectral interval was established from 100 to 1400 cm-1. The spectra were analyzed using the Opus 6.5 software (Bruker Optics, Ettlingen, Germany), and 12-point readings were randomly determined for 5 seconds in each spectrum. The spectral data of UA sealers were calculated and plotted together with other medium spectra of the same sealer for comparisons. Two examiners not involved in spectrum acquisition conducted the analyses.

### Statistical Analysis

Data were recorded for statistical evaluation using SPSS software version 23.0 (IBM Corp, Armonk, New York, USA). One-way ANOVA was used to analyze pH, calcium ion release, setting time, and solubility. The Tukey multiple comparisons test was used for the analyses of pH, calcium ion release, and solubility. The level of significance was set at 5%. The result of Raman spectroscopy was analyzed descriptively.

## Results

Table 1 presents the results for initial and final setting times, and solubility.

UA of sealers did not have any significant effect on the initial and final setting times, and solubility of groups (p>0.05). However, BCS and BR sealers had the highest solubility values in both activated and non-activated groups (p<0.05).

**Table 1:** Mean and standard deviations of initial and final setting times (min), and solubility

Initial Setting Time			
Without activation		Ultrasonic activation	
SP	102.00±3.00^a^	SP/UA	107.00±1.73^a^
SPBC	178.67±2.30^a^	SPBC/UA	162.67 ±12.70^a^
BCS	136.00 ±0.00^a^	BCS/UA	124.67±1.52^a^
EBC	219.33±3.05^a^	EBC/UA	195.00±3.00^a^
BR	114.67±10.26^a^	BR/UA	106.67±4.61^a^
Final Setting Time			
Without activation		Ultrasonic activation	
SP	114.00±0.00^a^	SP/UA	135.00±0.00^a^
SPBC	228.00±0.00^a^	SPBC/UA	180.33±20.20^a^
BCS	177.00±0.00^a^	BCS/UA	152.00±0.00^a^
EBC	238.33±3.51^a^	EBC/UA	222.33±2.51^a^
BR	149.00±9.53^a^	BR/UA	120.33±4.04^a^
Solubility			
Without activation		Ultrasonic activation	
SP	2.06±0.85^Aa^	SP/UA	1.09±0.51^Aa^
SPBC	0.84±0.10^Aa^	SPBC/UA	0.93±0.55^Aa^
BCS	20.82±2.^07Ba^	BCS/UA	18.00±2.82^Aa^
EBC	3.30±2.49^Aa^	EBC/UA	3.48±0.37^Aa^
BR	17.51±3.56 ^Ba^	BR/UA	16.42±1.13^Aa^

The same capital letters in the column indicate that values do not differ statistically.

The same lowercase letters in the line indicate that values do not differ statistically.

Significance level = 5%.

Table 2 presents the results for pH and calcium ion release for both groups at all time points.

All calcium silicate sealers had significantly higher pH values than SP and SP/UA from 24 hours onwards (p< 0.05). BR and BR/UA had significantly higher pH values than the other sealers at all time points (p< 0.05). Intragroup analyses revealed that the epoxy resin-based sealers, SP and SP/UA, had stable pH values over time (p>0.05); while calcium silicate sealers had increasing pH values (p<0.05). The comparison of pH values between activated and non-activated sealers revealed higher pH values for UA sealers, but the difference was not statistically significant (p>0.05).

**Table 2:** Mean values and standard deviations of pH and Ca+ release (mg/L) at the different time points.

pH measurements				
Sealers	1h	24h	72h	168h
SP	6.53±0.74^Ac^	7.28±0.55^Ad^	7.11±0.29^Ac^	7.04±0.72^Ac^
SP/UA	6.83±0.47^Ac^	7.55±0.15^Ad^	7.48±0.42^Ac^	7.57±0.26^Abc^
SPBC	6.42±0.26^Cc^	9.69±0.26^Bb^	10.03±0.22^Ab^	10.36±0.36^Ab^
SPBC/UA	6.80±0.86^Cc^	9.70±0.53^Bb^	10.10±0.60^Ab^	10.43±0.89^Ab^
BCS	6.22±0.35^Bc^	9.29±0.49^Ac^	10.00±0.28^Ab^	10.17±1.02^Ab^
BCS/UA	6.86±0.41^Cc^	9.37±0.26^Bc^	10.46±0.41^Ab*^	10.54±0.59^Ab^
EBC	8.15±1.01^Bb^	10.15±0.19^Ab^	10.24±0.36^Ab^	10.77±0.28^Ab^
EBC/UA	8.18±0.92^Bb^	10.79±0.29^Ab^	10.86±0.33^Ab*^	10.88±0.42^Ab^
BR	10.90±0.08^Ca^	11.36±0.41^Ba^	11.82±0.19^Aa^	11.84 ±0.24^Aa^
BR/UA	11.11±0.24^Ba^	11.79±0.12^Aa*^	11.87±0.15^Aa^	11.89±0.11^Aa^
Calcium ion release measurements				
Sealers	1h	24h	72h	168h
SP	15.66±17.34^Aa^	25.62±8.98^Aa^	23.97±18.73^Aa^	58.30±36.45^Ba^
SP/UA	26.50±14.10^Aa^	29.94±8.11^Aa^	45.06±29.96^Aa^	35.27±17.91^Aa^
SPBC	288.97±27.16^Ab^	419.78±61.23^Bb^	496.18±66.03^Bb^	498.08±11.48^Bb^
SPBC/UA	309.32±88.69^Ab^	434.37±29.01^Ab^	436.31±32.22^Ab^	477.41±97.33^Ab^
BCS	278.18±44.65^Ab^	281.16±99.58^Ab^	366.37±97.50^Bb^	459.96±59.04^Bb^
BCS/UA	276.03±48.46^Ab^	335.64±34.41^Ab^	509.08±173.35^Bb^	543.58±154.05^Bb^
EBC	400.64±136.82^Ab^	430.22±54.56^Ab^	481.68±201.84^Ab^	608.09±146.45^Ab^
EBC/UA	439.10±116.02^Ab^	767.79±136.67^Bc*^	766.13±94.50^Bc*^	787.76±181.86^Bc*^
BR	578.11±80.80^Ac^	652.29±229.62^Ac^	747.92±57.04^Ac^	767.27±40.27^Ac^
BR/UA	637.90±94.62^Ac^	796.51±88.99^Ac^	743.78±55.84^Ac^	764.58±41.54^Ac^

The same capital letters in the line indicate that values do not differ statistically.

The same lowercase letters in the column indicate that values do not differ statistically.

* indicates the statistical difference between UA and not activated sealers (significance level 5%).

SP and SP/UA had the lowest calcium release values at all time points (p<0.05) UA did not significantly affect calcium release of epoxy resin-based sealers (p>0.05). An increased calcium ion release was observed during intragroup analysis, however, only EBC and EBC/UA had a significant difference in calcium ion release at 24, 72, and 168 hours (p<0.05).

**Figure 1:**
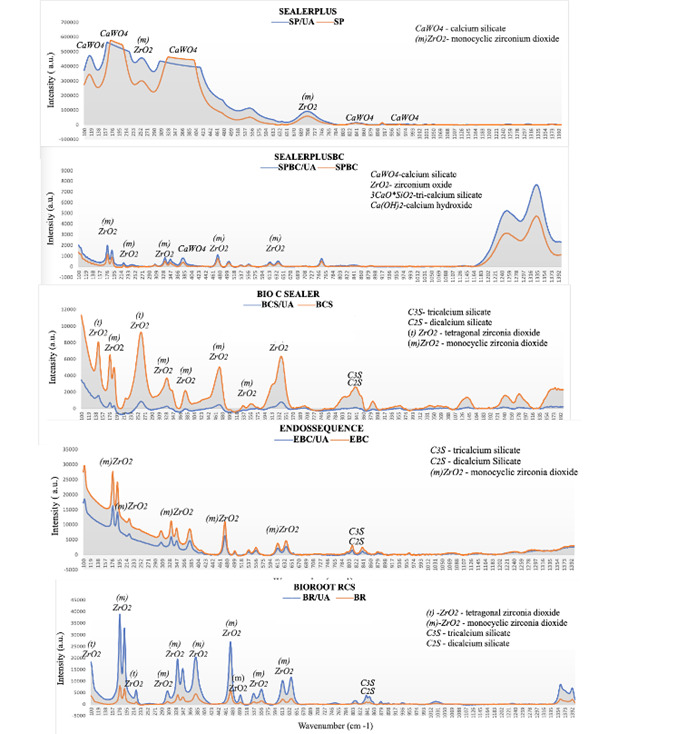
Raman Spectroscopy results depicting the chemical structure of the investigated sealers with and without ultrasonic activation (UA).

Raman spectroscopy results revealed the presence of calcium zirconia dioxide (ZrO2), dicalcium silicate (C2S), and tricalcium silicate (C3S), as well as monocyclic zirconia dioxide (m-ZrO2) in calcium silicate sealers. Additionally, BCS had two crystalline phases of zirconia dioxide: tetragonal and monocyclic (t-ZrO2; m-ZrO2), and SP had monocyclic zirconia dioxide (m-ZrO2) and calcium tungstate (CaWO4) (Figure 1).

## Discussion

As there is scarce information about the effects of ultrasonic activation on the physicochemical properties and chemical structure of calcium silicate-based sealers, this study was conducted to determine whether UA could impact setting time, solubility, pH, and calcium ion release, and chemical structure of calcium silicate-based sealers.

Previous studies that evaluated sealer ultrasonic activation employed activations for 10 or 20 seconds ^7^ ^,^ ^8^ ^,^ ^9^ ^,^ ^12^. In this study, an activation of 60 seconds was chosen to evaluate the sealers after an extended ultrasonic activation. As a control group, Sealer Plus (SP), an epoxy resin-based sealer, was chosen due to its good physicochemical properties ^16^.

BR and BR/UA had the shortest final setting time in all experimental conditions, probably because of the aqueous vehicle in its composition, which accelerates the chemical reactions of the sealer setting, as corroborated by previous studies ^12^ ^,^ ^17^. A slow setting time may clinically affect the periapical tissues, as most sealers produce some degree of toxicity until their final setting time and may be prone to solubility, which could lead to voids in the obturation ^5^.

All sealers that underwent UA had higher pH levels at all experimental time points, but intragroup comparisons found no significant differences. BR and BR/UA had higher pH levels than the other sealers at all time points. This data is corroborated by a previous study ^12^ that also ultrasonically activated this sealer. This might be explained by its high ionic dissociation in the aqueous vehicle.

The values of calcium release for SP and SP/UA samples were the lowest at all time points and when compared with the other sealers. These results agree with a previous study that found a low calcium release value for epoxy resin-based sealers when compared to calcium silicate-based sealers ^16^. Both BR and BR/UA had higher calcium ion release values than sealers in all the other groups, which is also in agreement with previous studies ^12^ ^,^ ^18^ that found higher calcium ion release for BR at all time points. These results may be explained by the substantial ionic diffusion found in an aqueous vehicle. Additionally, calcium ion release was significantly increased for EndoSequence BC Sealer when ultrasonically activated. It could be hypothesized that this is related to the extended final setting time of the sealer compared to the other evaluated sealers ^19^, allowing a greater ionic dissociation when ultrasonically activated. However, future studies are necessary to confirm this hypothesis.

There were no statistical differences in setting times between activated and not-activated sealers. Controversially, Ames et al. ^12^ reported that UA progressively delayed initial and final setting times for all calcium silicate sealers tested (BCS, SPBC, and BR). A plausible explanation for the outcome of this study is the fact that slightly moistened pieces of gauze were placed on the samples to provide SPBC, BCS, and EBC with the moisture needed to achieve their final setting times. Although it is not possible to measure the amount of water incorporated, this may have been an important source of moisture for the tested sealers.

Calcium silicate sealers, such as BCS and SPBC, have thickeners without water and are, therefore, sold as premixed sealers ready for use. This, however, makes the final setting time of these sealers dependent on the presence of moisture inside the dentinal tubules ^20^. The manufacturers do not recommend over-drying the root canals with absorbent paper cones, because the amount of moisture in the dentinal tubules and canal walls may be affected ^21^. A smear layer, or even tubular sclerosis, may also affect this property ^22^. 

Previous studies have demonstrated that the solubility of calcium silicate sealers is higher than in epoxy resin-based sealers ^23^ ^,^ ^24^ ^,^ ^25^. A possible explanation for that may be the fact that moisture may change the characteristics of hydrophilic materials ^5^. Additionally, solubility may be associated with differences in processes during setting, such as water absorption during this step. Although water absorption produces calcium hydroxide by hydration, the high potential for water absorption and solubility of calcium silicate sealers may reduce their dimensional stability and negatively affect their sealing ability ^5^. 

Although ultrasonic activation might promote temperature increase and, therefore, could heat the sealer which could promote chemical alterations, in this study, it did not affect the chemical structure of the investigated sealers. Based on the results observed by Raman spectroscopy, the peaks of the chemical bonds remained unchanged. Additionally, the plateaus observed may be due to the detector's saturation and there might have been some noises in signal reading or integration during analysis.

Ultrasonic activation of calcium silicate sealers may be a useful strategy for clinical practice. The results of this study did not find any negative impacts of ultrasonic activation on setting time, pH and calcium ion release, solubility, and chemical structure of any of the investigated sealers. Nevertheless, it is important to notice that the present study is limited to in vitro investigations, and conditions may vary during clinical applications.

## Conclusion

Based on the results of the present study, it can be concluded that ultrasonic activation did not significantly affect the initial and final setting times, solubility, pH, and chemical structure of the investigated sealers. Calcium ion release was significantly increased only for EndoSequence BC Sealer when ultrasonically activated.
